# Deep sequencing of small RNAs reveals the repertoire of miRNAs and piRNAs in *Biomphalaria glabrata*

**DOI:** 10.1590/0074-02760190498

**Published:** 2020-07-01

**Authors:** Fábio Ribeiro Queiroz, Laysa Gomes Portilho, Wander de Jesus Jeremias, Élio Hideo Babá, Laurence Rodrigues do Amaral, Luciana Maria Silva, Paulo Marcos Zech Coelho, Roberta Lima Caldeira, Matheus de Souza Gomes

**Affiliations:** 1 Fundação Oswaldo Cruz-Fiocruz, Instituto René Rachou, Grupo de Pesquisa em Biologia do Schistosoma mansoni e sua Interação com o Hospedeiro, Belo Horizonte, MG, Brasil; 2 Universidade Federal de Uberlândia, Laboratório de Bioinformática e Análises Moleculares, Patos de Minas, MG, Brasil; 3 Universidade Federal de Ouro Preto, Escola de Farmácia, Departamento de Farmácia, MG, Brasil; 4 Fundação Ezequiel Dias, Serviço de Biologia Celular do Departamento de Pesquisas e Desenvolvimento, Belo Horizonte, MG, Brasil; 5 Fundação Oswaldo Cruz-Fiocruz, Instituto René Rachou, Grupo de Pesquisa em Helmintologia e Malacologia Médica, Belo Horizonte, MG, Brasil; 6 Universidade Federal de Uberlândia, Rede Multidisciplinar de Pesquisa, Ciência e Tecnologia, Patos de Minas, MG, Brasil

**Keywords:** schistosomiasis, non-coding RNAs, mollusk vectors, next-generation sequencing

## Abstract

**BACKGROUND:**

*Biomphalaria glabrata* snails are widely distributed in schistosomiasis endemic areas like America and Caribe, displaying high susceptibility to infection by *Schistosoma mansoni*. After the availability of *B. glabrata* genome and transcriptome data, studies focusing on genetic markers and small non-coding RNAs have become more relevant. The small RNAs have been considered important through their ability to finely regulate the gene expression in several organisms, thus controlling the functions like cell growth, metabolism, and susceptibility/resistance to infection.

**OBJECTIVE:**

The present study aims on identification and characterisation of the repertoire of small non-coding RNAs in *B. glabrata* (Bgl-small RNAs).

**METHODS:**

By using small RNA sequencing, bioinformatics tools and quantitative reverse transcription polymerase chain reaction (RT-qPCR), we identified, characterised, and validated the presence of small RNAs in *B. glabrata*.

**FINDINGS:**

89 mature miRNAs were identified and five of them were classified as Mollusk-specific. When compared to model organisms, sequences of *B. glabrata* miRNAs showed a high degree of conservation. In addition, several target genes were predicted for all the mature miRNAs identified. Furthermore, piRNAs were identified in the genome of *B. glabrata* for the first time. The *B. glabrata* piRNAs showed strong conservation of uridine as first nucleotide at 5’ end, besides adenine at 10th position. Our results showed that *B. glabrata* has diverse repertoire of circulating ncRNAs, several which might be involved in mollusk susceptibility to infection, due to their potential roles in the regulation of *S. mansoni* development.

**MAIN CONCLUSIONS:**

Further studies are necessary in order to confirm the role of the Bgl-small RNAs in the parasite/host relationship thus opening new perspectives on interference of small RNAs in the organism development and susceptibility to infection.

The *Biomphalaria sp glabrata* snails are the most important intermediate hosts in the transmission of *Schistosoma mansoni*, one of the main causative agents of hepatosplenic form of schistosomiasis in America and Caribe. The disease affects millions of people worldwide and, in Brazil its transmission is maintained mainly by *B. glabrata*, which has a wide geographic distribution in endemic areas, showing high susceptibility to infection by *S. mansoni*.^1^ The interaction between parasite and mollusk is complex and well described in many aspects.^2^ However, the genetic mechanisms that closely explain this interaction are poorly understood. The habits of the human population, the snail distribution, and the absence of basic sanitation, make schistosomiasis very difficult to be eradicated through the interruption of the parasite life cycle. Furthermore, one of the factors that influence the susceptibility of the snail to infection by *S. mansoni*, is the activity of its internal defense system (IDS), which relies on hemocytes and soluble components of hemolymph.^3^ ^,^ ^4^ Studies showed that transferring the hemolymph from snails resistant to *S. mansoni* infection to susceptible ones increases the resistance of the later.^5^ These results brought to light the question whether is small RNA part of the genetic mechanisms involved in controlling the activity of the hemocytes, and soluble factors of hemolymph, which are determinants of the susceptibility/resistance of the snail to infection by the trematode.

After the availability of the genome and the transcriptome data of *B. glabrata*,^6^ the studies with focus on genetic markers have become more relevant. Our research group identified genes from the small RNA pathway of different developmental stages of *B. glabrata*, and changes on their expression profiles during the infection by *S. mansoni*.^7^ The miRNA expression profile and its pathway have been extensively described in *S. mansoni*,^8^ ^,^ ^9^ ^,^ ^10^ but in *B. glabrata* the studies directed towards the identification and characterisation of these small RNAs are still poorly explored.

Small RNAs and their silencing pathways have been considered important in several organisms, since they orchestrate gene expression through a fine and specific regulation process.^11^ Some of the most important small RNAs are microRNAs (miRNAs) and PIWI-interacting RNAs (piRNAs), which differ in the number of nucleotides, biogenesis pathway, biological origin and functions, and target genes. Typically, miRNAs have 17 to 24 nucleotides, while in piRNAs, the nucleotides range between 23 to 35.The miRNAs are the best-characterised class among all small RNAs, and their sequences and features can be found in the main database containing thousands of miRNAs from several species (miRBase - http://www.mirbase.org/). Another class of endogenous small RNAs, piRNAs, are longer and their active sequences are more diverse when compared to miRNAs. The sequences and features of piRNAs are studied using databases like piRNABank (http://pirnabank.ibab.ac.in/) and piRBase (http://regulatoryrna.org/database/piRNA).

The biogenesis of miRNAs is well studied, and it relies on nuclear and cytoplasmic proteins. Normally, the mature miRNAs recognise the mRNA targets 3’ UTR region and the pairing does not need to be perfect for silence activity.^12^ The association between these molecules causes blockade of translation or mRNA degradation, reducing this way levels of the protein coded by a specific gene target.^12^ The piRNAs can be originated from transposable elements and genomic clusters and, their main functions are closely related to transposons control, mainly on germ line cells.^13^ ^,^ ^14^ ^,^ ^15^ However, it is a currently well-known fact that piRNAs are involved in the development of somatic tissues such as, neurons, muscle and adult stem cells.^16^ ^,^ ^17^ The piRNAs have a notable diversity in sequences, except in the first nucleotide at 5’ end, frequently one uridine, beyond an adenine on the tenth position.^13^ The biogenesis of the piRNA is not well known as that of miRNAs, and it can be divided into two steps: transcription of long precursors inside the nucleus, followed by their exportation to the cytoplasm, where it is processed to generate mature piRNAs. The mature piRNAs associated with Piwi proteins act on repression of translation or cleavage of mRNAs targets.^18^ In animals, besides the canonical biogenesis of the piRNAs, another common pathway is the “ping-pong cycle”, that further shapes the piRNA population by amplifying sequences targeting active transposons.^13^ On the cytoplasm, precursors of piRNAs exported from nucleus are processed without dicer and in association with AGO-like proteins, act to control gene expression and transposons activities.^19^

Advances in high-throughput sequencing technologies have significantly increased the understanding of the role of small RNAs on several species, including *B. glabrata*. The genome and transcriptome sequencing^6^ revolutionised the knowledge of the relationship between the snail and *S. mansoni* by bringing to light the existence of processing machinery of small RNAs such as miRNAs and piRNAs, in *B. glabrata*.^6^ ^,^ ^7^ However, it is necessary to identify and characterise the profile of miRNAs and piRNAs expression within this organism*.* In the present work our group applied high-throughput sequencing technologies and bioinformatics tools to reveal the expression profiles of these small RNAs, which allowed us to predict new insights on their involvement in the biology of *B. glabrata*.

## MATERIALS AND METHODS

*Biological samples and RNA preparation* - *B. glabrata* mollusks (Belo Horizonte strain - 056/2012/SECEX/CGEN) were obtained from Moluscario Lobato Paraense at the René Rachou Institute. For small RNA sequencing, 10 snails with 12 to 15 mm in diameter, were pooled for each group. The groups, with shell and without shell, were chosen to represent, respectively, mollusks with whole hemolymph and with poor amount of hemolymph. For quantitative polymerase chain reaction (qPCR), egg masses and snails at different developmental stages (10, 20, and 40 days) were used. RNA preparations were made from triplicates of 10 snails per specific stage. Total RNA was isolated and used for sequencing and qPCR assays. The samples were frozen in liquid nitrogen, macerated and homogenised with Tri Reagent^®^ (Sigma-Aldrich). The total RNA was finally treated with DNase (TURBO DNA-free kit Ambion^®^), as recommended by the manufacturer and quantified by using Nano Drop nano-spectrometer. The RNA quality was analysed through capillary electrophoresis on Bioanalyzer (Agilent^®^). cDNA synthesis was determined by High-capacity Kit (Life Technologies™) according to manufacturer’s instructions.

*Small RNA sequencing and quantitative reverse transcription polymerase chain reaction (RT-qPCR)* - The small RNA fraction was purified from total RNA of *B. glabrata* and used afterward for library construction and sequencing through Illumina HiSeq^TM^ 2500 system according to manufacturer’s instructions. The sequences are available in the SRA database (PRJNA596001). For qPCR, TaqMan^®^system (Life Technologies^TM^) was chosen and the assays were performed on ViiA 7^TM^ Real-Time PCR System (Applied Biosystems^TM^). The TaqMan^®^assay oligonucleotides were manufactured by Life Technologies™ [Supplementary data **(Table I)**]. The miRNAs expression level was measured in three biological replicates of snail samples from each different age and, gene expression levels were normalised by using the U6 transcript as internal control. The relative quantification of the expressed gene was determined by the 2^-ΔΔCt^ method^20^ and statistical analyses among the different groups was performed by using the One-Way ANOVA, with Tukey test as *post-hoc* test on GraphPad Prism^®^ 5.0 (GraphPad Software, Inc., San Diego, USA). Values of p ≤ 0.05 were considered statistically significant and are denoted with asterisks in the figures.

*Small RNA sequencing data* - The reads sequenced from the different samples of *B. glabrata* adult snails, with and without shell, were filtered according to the quality scores by using the FastQC software and the adapters sequences trimmed through Trimmomatic software (http://www.usadellab.org/cms/?page=trimmomatic). Reads with length ranging from 15 to 35 nucleotides were selected and, those considered with low quality (quality score < 25) were discarded from the study.

*miRNA and piRNA analyses* - All valid read sequences were counted and aligned onto *B*. *glabrata* genome (https://www.vectorbase.org/organisms/biomphalaria-glabrata) by using bowtie andmiRDeep2 software (https://github.com/rajewsky-lab/mirdeep2). Precursor miRNA sequences used as reference for the analyses were obtained from Adema, 2017.^6^ Beside this, the mature sequences used in the NGS analysis were also predicted from the alignment between the putative *B. glabrata* precursor miRNAs and their respective ortholog from animal species retrieved from miRBase database (http://www.miRbase.org/), accepting until five mismatches.

For the identification of miRNA target genes, we collected 3′-UTR-sequences from the annotation features defined in the GFF3 file from *B. glabrata* genome. Furthermore, in order to search for the gene targets, miRanda software (http://www.microrna.org/) and RNAhybrid software (http://bibiserv.techfak.uni-bielefeld.de/rnahybrid) were used. The miRanda software was set with the following parameters and conditions: a gap opening penalty of -8, a gap extension penalty of -2; match with minimum score threshold 120, target duplex with maximum threshold free energy -15 kcal/mol, scaling parameter three for complementary nucleotide match score, counting from the miRNA 5′ end, and demand strict 5′ seed pairing between two and nine nucleotides. The RNAhybrid software was also used for the same purpose and it was set with default parameters. Only the miRNA target genes found in both software were considered for further analysis.

For the piRNA analysis, only reads with length between 23 and 35 nucleotides were included. Reads mapped onto Bgl-miRNAs and Bgl-rRNA sequences were filtered and removed from the study in this step. Predicted clusters of Bgl-piRNAs were obtained by using proTRAC software set according to the pipeline suggested by Rosenkranz. The ping-pong cycle signature was predicted by using PingPongPro software (https://sourceforge.net/p/pingpongpro/), properly set to a predefined length of putative Bgl-piRNAs.

## RESULTS

**Fig. 1:**
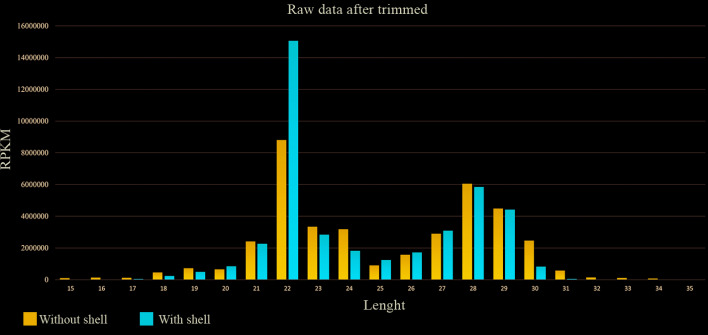
raw data after trimmed. After trimming, reads sequences from *Biomphalaria glabrata* with and without shell revealed size distribution ranging from 21 to 24 and 27 to 30 suggesting a profile of miRNAs and piRNAs, respectively.

The small RNA sequencing for both samples, with and without shell, showed a large amount of reads with great diversity and quantity. The counts of unique and total reads suggested this diversity (Table I) with large differences for both samples. Sequences of 15 to 35 nucleotides were kept for small RNAs analysis and over 43 million reads were obtained from samples without shell, while more than 26 million from samples with shell. The two groups showed similar distribution for read sizes, with typical length of the miRNAs sequences, ranging from 21 to 24 nucleotides and for piRNAs from 27 to 30 nucleotides (Fig. 1).

**TABLE I:** Small RNA sequencing counts of reads

	Without shell		With shell	
	Unique	Total	Unique	Total
Reads (Adapter trimmed)	1123762	44103293	2785427	26488178
Clean reads (15-35 nt)	968931	43290179	2728664	26404729
Not matching genome	624307	12344582	1759485	7598633
Matching genome	344624	30945597	969179	18806096
Matching mature core miRNAs	86	12629502	89	10236064
Matching piRNAs	33377	2696565	209957	3954541
Matching Rfam database	210527	17032569	251260	1938236
tRNA	117015	15635721	87557	972458
rRNA	73865	1192639	124043	718291
snoRNA	1068	5195	2394	22094
snRNA	762	22015	960	35491
other	17817	176999	36306	189902
Unknown reads	879772	11744657	2324121	10359337

Initially, the raw data was compared with sequences on Rfam database, which allowed identification of small RNAs [Supplementary data **(Fig. 1)**] presented in the samples. Sixty eight pre-miRNAs annotated in the *B. glabrata* genome [Supplementary data **(Table II)**] were identified as precursors^6^ for 89 mature miRNAs identified in the small RNA sequencing samples (Table II).

**TABLE II:** Characterisation of mature miRNAs on *Biomphalaria glabrata*

Mature miRNA name	Mature sequence	Mature miRNA size	Start-End position in precursor	Orthologue mature miRNA	Mismatches with orthologue miRNA	Number of reads in NGS	
						Without shell	With shell
bgl-bantam-3p	ugagaucauugugaaaacugauu	23	47-69	ame-bantam	1	113854	1307
bgl-bantam-5p	cugguuuucccauuggucuggcag	24	10-33	tca-bantam-5p	5	8	97
bgl-let-7-5p	ugagguaguagguuguauuguu	22	2-23	cel-let-7-5p	1	156393	2006275
bgl-miR-100-3p	acaaguuugcaucuauggguaug	23	58-80	tgu-miR-100-3p	4	27	13
bgl-miR-100-5p	aacccguagaaccgaacuugugc	23	17-39	oha-miR-100-5p	1	4489426	12675
bgl-miR-1175-3p	ugagauucaacuccuccaacugc	23	59-81	cte-miR-1175-3p	0	8517	769
bgl-miR-1175-5p	aguggagagaguuuuaucucau	22	18-39	cte-miR-1175-5p	0	1037	921
bgl-miR-124-3p	uaaggcacgcggugaaugccaag	23	57-79	dme-miR-124-3p	0	347	2440
bgl-miR-124-5p	cgcguucacugggucagccuug	22	20-41	tur-miR-124-1-5p	5	0	1
bgl-miR-125-5p	ucccugagaccauaauuugug	21	20-40	dme-miR-125-5p	2	17076	57332
bgl-miR-12-5p	ugaguauuacuucagguacug	21	10-30	bmo-miR-12	0	81532	2164
bgl-miR-1260-3p	aucccaccgcugccacca	18	50-67	cgr-miR-1260	0	13	273
bgl-miR-133-3p	uuugguccccuucaaucaguugua	24	57-80	efu-miR-133-3p	2	6334	24934
bgl-miR-133-5p	agcugguugaaucugggccaaau	23	17-39	lgi-miR-133-5p	2	1916	13
bgl-miR-137-3p	uuauugcuugagaauacacgua	22	52-73	ame-miR-137	0	147	179
bgl-miR-137-5p	acggguauucuuggguaaauaau	23	15-37	oha-miR-137-5p	1	0	9
bgl-miR-153-3p	uugcauagucacaaaagugauc	22	59-80	mmu-miR-153-3p	0	1970	70
bgl-miR-184-3p	acuggacggagaacugauaagggc	24	72-95	bmo-miR-184-3p	0	2185751	2402352
bgl-miR-190-5p	agauauguuugauauauuuggugg	24	18-41	cte-miR-190	0	3534	584
bgl-miR-193-3p	uacuggccuucaaaaucccaa	21	52-72	lgi-miR-193	1	1137	3766
bgl-miR-1984-5p	ugcccuauccgucaggaacugug	23	9-31	hru-miR-1984	0	403721	144101
bgl-miR-1985-5p	ugccauuuuuaucagucacugug	23	17-39	hru-miR-1985	0	127450	14703
bgl-miR-1986-3p	uggauuucccaagauccgugau	22	57-68	hru-miR-1986	0	40	7522
bgl-miR-1990-3p	cgggacuacgucaacu	16	56-71	cte-miR-1990c-3p	2	637	1228
bgl-miR-1990-5p	aguaaguugauggggucccagg	22	20-41	hru-miR-1990	0	144	392
bgl-miR-1991-5p	cuuacccuguuauacugagaagu	23	16-38	hru-miR-1991	3	541	114
bgl-miR-1992-3p	ucagcaguuguaccacugauuug	23	59-81	lgi-miR-1992	0	180	1255
bgl-miR-1993-3p	uauuaugcugcuauucacgaga	22	45-66	cte-miR-1993	1	432	160
bgl-miR-1994a-3p	ugagacaguguguccucccuug	22	53-74	lgi-miR-1994a	0	1053	779
bgl-miR-1994b-3p	ugagacagugcguccucccuca	22	51-72	lgi-miR-1994b	1	945	4911
bgl-miR-199a-3p	gcaguagucugcacauuguuua	22	62-83	mmu-miR-199a-3p	2	0	1
bgl-miR-1a-3p	uggaauguaaagaaguauguau	22	69-90	mmu-miR-1a-3p	0	978	3867163
Bgl-miR-1a-5p	acauacuucuuugcuaucccau	22	32-53	mmu-miR-1a-5p	4	7	572
bgl-miR-2001-5p	uugugaccguuauaaugggcauu	23	20-42	lgi-miR-2001	0	11799	2051
bgl-miR-216a-5p	uaaucucagcugguaauucagag	23	10-32	lgi-miR-216a	1	3141	123
bgl-miR-216b-5p	uaauaucagcugguaauccugag	23	9-31	lgi-miR-216b	0	25179	804
bgl-miR-219a-3p	agaacuguguguggacaucagu	22	50-69	ipu-miR-219a	4	46	41
bgl-miR-219a-5p	ugauuguccaaacgcaauucuug	23	22-44	ssa-miR-219a-5p	0	51	269
bgl-miR-252a-5p	cuaaguacuggugccgcggga	21	24-44	lgi-miR-252a	0	16404	40203
bgl-miR-252b-3p	accugcacaccggugcuua	19	55-73	str-miR-252b-3p	3	4	1
bgl-miR-252b-5p	auaaguaguggugccgcaggua	22	18-39	lgi-miR-252b	0	986	35027
bgl-miR-2722-3p	uggcgccguggaaacaucuacc	22	51-72	lgi-miR-2722	0	685	132
bgl-miR-277a-3p	uaaaugcauuaucugguaucu	21	60-80	cte-miR-277a	1	79902	7332
bgl-miR-278-3p	ucggugggacuuucguucguuu	22	62-83	sko-miR-278	0	25242	102
bgl-miR-279-3p	ugacuagauccacacucaucca	22	55-76	lgi-miR-279	0	50935	35566
bgl-miR-279-5p	gauggcuguuggucuggugcaug	23	19-41	tca-miR-279b-5p	4	488	1168
bgl-miR-281-3p	acugucauggaguugcucucuu	22	42-63	bmo-miR-281-3p	0	4685	39
bgl-miR-281-5p	aagggagcauccgucgacagu	21	9-29	lgi-miR-281-5p	1	126826	4808
bgl-miR-29a-3p	uagcaccauuugaaaucaguuu	22	62-83	cte-miR-29a	0	553	3206
bgl-miR-29b-3p	uagcaccauuugaaaucaguuu	23	55-77	bfl-miR-29b-3p	0	553	3206
bgl-miR-2a-1-3p	uaucacagccagcuuugaugagcg	24	54-77	api-miR-2a	0	7195	11517
bgl-miR-2a-2-3p	uaucacagccagcuuugauga	21	52-72	dme-miR-2a-3p	0	15098	46019
bgl-miR-2a-2-5p	cgucaaggcgguugugaugug	21	16-36	asu-miR-2a-5p	4	206	577
bgl-miR-2b-3p	uaucacagucagcuuugaugagcu	24	57-81	aae-miR-2b	1	15098	13687
bgl-miR-2d-3p	uaucacagccugcuuggaucagu	23	53-75	lgi-miR-2d	0	14506	4997
bgl-miR-315-5p	uuuugauuguugcucagaaagcc	23	11-33	cte-miR-315	0	1607001	30357
bgl-miR-317-3p	ugaacacagcuggugguaucuuau	24	56-79	lgi-miR-317	1	46000	14531
bgl-miR-33-3p	caaugucucugcagugcaau	20	52-71	oan-miR-33a-3p	2	160	18
bgl-miR-33-5p	gugcauuguaguugcauugcgug	23	18-40	tca-miR-33-5p	1	218	808
bgl-miR-34-3p	caaccacucuccacauuaccgcc	23	51-73	aae-miR-34-3p	5	54	29
bgl-miR-34-5p	uggcagugugguuagcugguugu	23	17-39	dme-miR-34-5p	0	7617	21912
bgl-miR-36a-3p	ucaccggguauacauucauccg	20	56-75	asu-miR-36a-3p	0	2	5
bgl-miR-375-3p	uuuguucguucggcucgcguuau	23	61-83	bbe-miR-375-3p	0	433042	774752
bgl-miR-67-1-5p	ucacaaccugcuugaaugaggac	23	23-45	lgi-miR-67	0	731134	52870
bgl-miR-67-2-3p	ucacaaccugcuugaaugaggac	23	50-72	lgi-miR-67	1	731134	52870
bgl-miR-71-5p	ugaaagacauggguagugagaug	23	18-40	cte-miR-71	0	10395	337118
bgl-miR-72-3p	agcugugucauauguugcca	20	52-71	str-miR-72-3p	4	19	75
bgl-miR-72-5p	aggcaagauguuggcauagcuga	23	25-37	cel-miR-72-5p	0	11838	15097
bgl-miR-7-3p	caauaaaucacaaucuuc	18	57-74	bbe-miR-7-3p	2	61	6
bgl-miR-745a-3p	agcugccugaugaagagcugu	21	55-75	lgi-miR-745a	0	23721	24336
bgl-miR-745b-3p	agcugccaaaugaagggcugu	21	49-69	lgi-miR-745b	0	64818	2126
bgl-miR-750-3p	ccagaucuaacucuuccagcuca	23	60-82	cte-miR-750	0	534947	9893
bgl-miR-750-5p	cguuggaggauuggaucuuagc	22	22-43	tca-miR-750-5p	5	2354	25
bgl-miR-7-5p	uggaagacuagugauuuaguuguu	24	18-41	ssc-miR-7	1	9	144
bgl-miR-8-3p	uaauacugucagguaaagauguc	23	63-85	dme-miR-8-3p	0	245404	47380
bgl-miR-8-5p	cgucuuaccuagcagcauugga	22	18-39	dvi-miR-8-5p	4	528	181
bgl-miR-87b-1-3p	gugagcaaaguuucagguguau	22	60-81	cte-miR-87b	0	2967	10380
bgl-miR-87b-2-3p	gugagcaaaguuucagguguau	22	59-80	cte-miR-87b	0	2967	10380
bgl-miR-92-3p	aauugcacucgucccggccugc	22	60-81	dpu-miR-92	0	651682	30648
bgl-miR-92a-1-3p	uauugcacuuuucccggccugu	22	57-78	hsa-miR-92a-3p	2	57848	11249
bgl-miR-92a-3p	uauugcacuuuuccaggccuuu	22	56-77	bfl-miR-92a	2	1048	2086
bgl-miR-92b-3p	aauugcacuaaucccggccuac	22	54-75	dme-miR-92b-3p	2	16497	41985
bgl-miR-96a-5p	cuuggcacuggcggaauaguca	22	10-31	lgi-miR-96a	1	5745	25313
bgl-miR-96b-5p	auuuggcacuuguggaauaaucg	23	16-38	lgi-miR-96b	0	7132	729
bgl-miR-981-3p	uucguugucgucgaaaccugccu	23	50-72	cte-miR-981	1	153740	15044
bgl-miR-9a-3p	auaaagcuagguuaccaaaggc	23	47-68	lgi-miR-9-3p	0	990	945
bgl-miR-9a-5p	ucuuugguuaucuagcuguauga	22	9-31	dme-miR-9a-5p	0	18353	817
bgl-miR-9b-3p	auaaagcuagguuaccaaaggc	22	47-68	lgi-miR-9-3p	0	990	945
bgl-miR-9b-5p	cuuugguaaccuagcuuuauga	22	1-23	ame-miR-9b	2	8	16

**Fig. 2:**
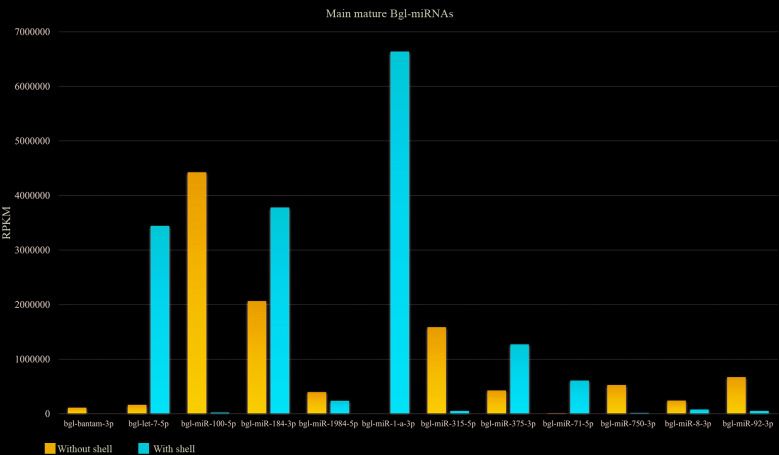
the most abundant Bgl-miRNAs in the samples sequenced. Mature miRNAs with larger counts of mapped reads in snails with shell and without shell.

The mature Bgl-miRNAs identified in small RNA sequenced libraries revealed a set of five Mollusk-specific miRNAs [Supplementary data **(Fig. 2)**]. In addition, some miRNAs stood out by presenting large reads counts, like bgl-bantam, bgl-let-7, bgl-miR-100, bgl-miR-184, bgl-miR-71, bgl-miR-1984, bgl-miR-1a, bgl-miR-315, bgl-miR-375, bgl-miR-750, bgl-miR-8 and bgl-miR-92. These mature Bgl-miRNAs represented 86% of total in the sample of snails without shell and 94% of those with shell (Fig. 2), suggesting these miRNAs may an important role in the snail biology and, for this, additional studies related to their putative targets were performed.

**Fig. 3:**
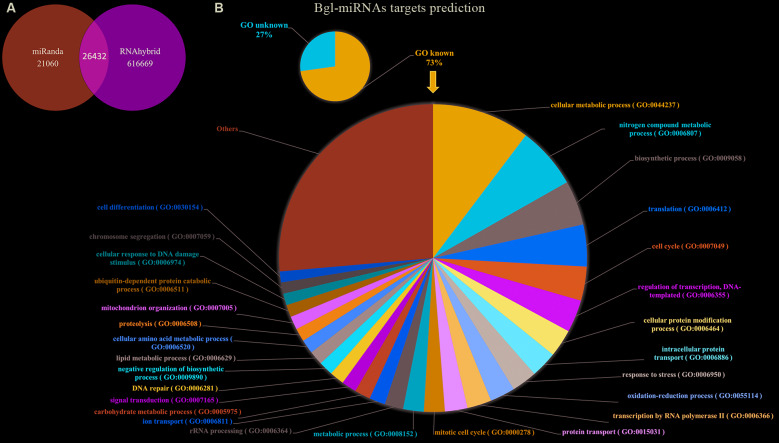
prediction of target-genes for Bgl-miRNAs in *Biomphalaria glabrata*. (A) 47492 putative targets found with miRanda software on *B. glabrata* 3´UTR se quences of genes, and 643101 with RNAhybrid. Only 26432 are common between them; (B) for the common predicted targets, 73% showed known GO. Those pertaining to the level of biological process were used to group the gene targets of Bgl-miRNAs.

*Identification of miRNA target genes in* B. glabrata - The strategy of using two softwares for prediction and, accepting as valid only those genes predicted by both, allowed the identification of several target genes for all the mature Bgl-miRNAs [Supplementary data **(Table III)**]. In order to improve the power of prediction of the results and decrease false positives, mismatches between the seed region of the mature Bgl-miRNA sequences and their respective 3´UTR target gene in *B. glabrata* genome were not accepted on both softwares. Over 40,000 predicted targets were identified by miRanda software and over 640,000 by RNAhybrid. However, only 26432 target genes were considered since they were identified by both softwares (Fig. 3A). Within these predicted targets, 73% represented known gene ontologies (GO) annotation, which supported the functional prediction of biological processes associated with miRNAs identified in this study (Fig. 3B).

*RT-qPCR validation* - After characterising *B. glabrata* miRNAs by high-throughput sequence method, the level of expression for the most outstanding miRNAs was measured by qPCR. A set of miRNAs including: bgl-miR-8, bgl-miR-92, bgl-miR-184, bgl-miR-315, bgl-miR-1984, bgl-miR-375 and bgl-miR-750 had their expression level evaluated in *B. glabrata* snails from different developmental stages by qPCR. Snails with 10, 20 and 40 days of age were selected as representative of different stages of the life cycle, including those sexually immature, in transition to adult stage and finally adult ones,^7^ Egg masses from *B. glabrata* were used as control.

**Fig. 4:**
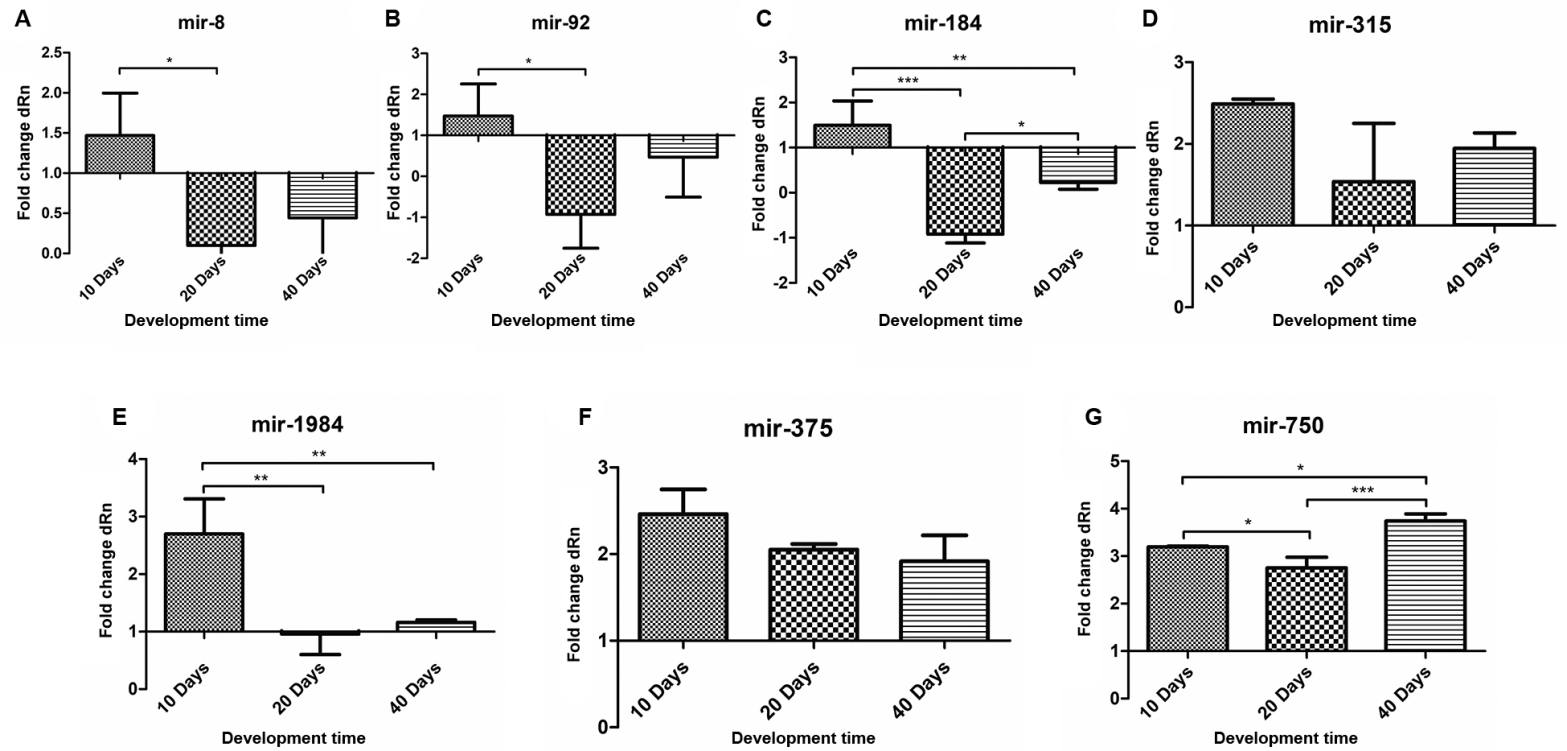
Bgl-miRNAs expression determined through quantitative reverse transcription polymerase chain reaction (RT-qPCR) in developmental stages of *Biomphalaria glabrata*. (A) bgl-miR-8 showed significant difference between 10 and 20 days. (B) bgl-miR-92 showed significant difference between 10 and 20 days. (C) bgl-miR-184 showed significantly different profile between all evaluated time. (D) bgl-miR-315 not presented nonsignificant difference between evaluated time. (E) blg-miR-1984 showed a significant over-expression when compared with 20 and 40 days. (F) bgl-miR-375 not presented nonsignificant difference between evaluated time. (G) bgl-miR-750 showed profile significantly different between all evaluated time.

There were significant differences in the miRNA expression in different stages of the snails (p < 0.05) (Fig. 4). This was more pronounced in the expression of bgl-miR-184 (Fig. 4C) and bgl-miR-750 (Fig. 4G). In age 20 and 40 days snails, bgl-miR-184 was under-expressed. However, there was an over-expression of bgl-miR-750 in snails of all ages analysed. Our results also showed thatbgl-miR-8 (Fig. 4A) and bgl-miR-92 (Fig. 4B) were significantly expressed in snails age 10 and 20 days. Similar observation was recorded for bgl-miR-184 expression. The bgl-miR-1984 (Fig. 4E) showed significant differences in its expression in 10, 20 and 40 days. No significant difference in expression was observed for bgl-miR-315 (Fig. 4D) and bgl-miR-375 (Fig. 4F) relative to snails ages.

*Identification of putative piRNAs on* B. glabrata - The piRNAs repertoire in *B. glabrata* showed a large count of reads with 28 to 29 nucleotides of length in both libraries of snails with and without shell [Supplementary data **(Fig. 3)**]. These sequences were compared, firstly, with Bgl-miRNA precursors and Bgl-rRNAs. Only reads unmapped onto those types of RNAs were considered for Bgl-piRNAs search.

**Fig. 5:**
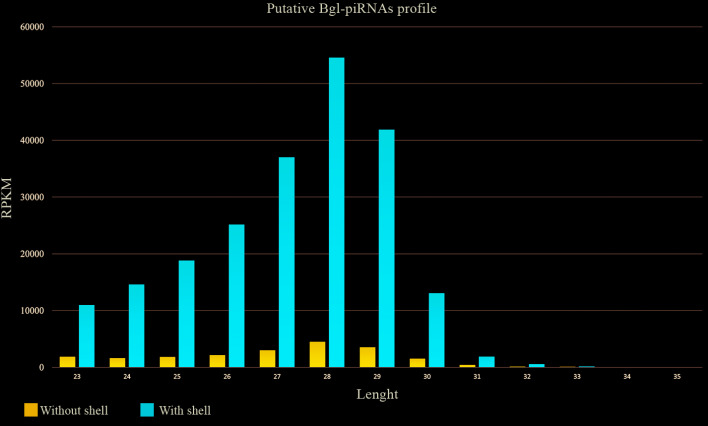
putative piRNA sequences in *Biomphalaria glabrata*. Distribution of putative Bgl-piRNAs separated by size, demonstrating a larger diversity of piRNAs at library of snail with shell in relation to library of snail without shell.

Filtered sequences corresponding to putative Bgl-piRNAs were analysed with ProTrac software (https://sourceforge.net/projects/protrac/) to predict piRNA clusters based on genome mapped piRNA sequence reads. The typical signatures of piRNAs, the presence of 1 U or 10 A on sequence, were used as attribute to infer the existence of classical piRNAs. It was found that 195 predicted clusters in the library constructed with samples of snails without shell [Supplementary data **(Table IV)]** comprise 49,155 putative piRNAs. A total of 352 predicted clusters in the library of snails with shell, comprised 314,729 putative piRNAs [Supplementary data **(Table V)**]. The sequences of putative piRNAs for both libraries were concatenated [Supplementary data **(Table VI)]** and the result of putative Bgl-piRNAs was presented as expected abundance of sequences with 27 to 29 nucleotides in length (Fig. 5).

**Fig. 6:**
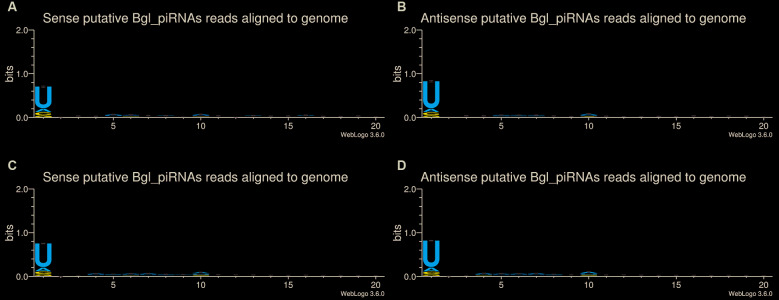
sequence logo of putative Bgl-piRNAs sequences. The sequence logo was generated by using the putative Bgl-piRNA sequences trimmed of 1 to 20 nucleotides. (A) sequence logo of strand sense of the samples without shell with remarkable uridine at first position. (B) sequence logo of strand antisense of the samples without shell with remarkable uridine at first position. (C) sequence logo of strand sense of the samples with shell with remarkable uridine at first position. (D) sequence logo of strand antisense of the samples with shell with remarkable uridine at first position.

Beyond the typical signature 1U-10A (Fig. 6), which is accordance with what is defined in other organisms as the ping pong cycle signature was also found in both libraries of snails with and without shell, further high scores for A at 10th position [Supplementary data **(Fig. 4)**]. Taken together, these findings reinforce the identification of piRNAs in *B. glabrata* genome.

## DISCUSSION

The susceptibility of *B. glabrata* to *S. mansoni* infection has been studied by different research groups around the world, some of whom attempted to unravel the genetic or physiologic mechanisms that regulate *B. glabrata* susceptibility to *S. mansoni* nfection. However, no study is currently available that elucidates the role of small RNAs in the snail host-parasite relationship. Our group has previously studied the expression profile of genes on miRNA biogenesis in the mollusk.^7^ In present the study, we were able to reveal numerous small RNAs expressed in different developmental stages of *B. glabrata* using a small RNA high throughput sequencing method and bioinformatics tools, thus predicting their involvement in regulating essential processes in the snail biology. The results presented in our study open the way to exploration of roles of small RNAs in *B. glabrata* development and susceptibility to infection by *S. mansoni*.

In this study, we used samples of the snails with and without shell. Snails without shell lose almost the whole amount of hemolymph during the process of shell removal. On the other hand, the samples comprising snails with shell keep their complete molecular contents. By using the two types of snails we could show differences between their repertoires of small RNAs, which suggested the importance of the hemolymph in the natural diversity of these molecules in *B. glabrata*. Previous studies have shown the importance of hemolymph as an essential factor to snail in fighting infection.^3^ ^,^ ^4^ ^,^ ^21^ The additional small RNAs observed in sample with preserved hemolymph are predicted to play additional functional roles in snail protection against *Schistosoma* infection.

When filtered to the range of 15 to 35 nucleotides, the sequenced reads with high quality revealed that the libraries presented a similar outline of small RNA lengths (Fig. 1). The reads found with 21 to 24 nucleotides in length are compatible with typical miRNAs mean size. These sequences were aligned onto the Rfam database [Supplementary data **(Fig. 1)**] and the highest counts contributed to enrich the miRNAs like when compared to piRNAs. Otherwise, the distribution of the reads between 27 to 30 nucleotides was compatible with piRNA sequences and, instead of the smaller counts per each sequence, a larger diversity of Bgl-piRNAs was found when compared to miRNAs and fragments of tRNA. The results of Bgl-piRNAs showed a larger diversity of sequences in snails with shell compared to those without shell, suggesting that the hemolymph of the *B. glabrata* has an expressive amount of circulating piRNAs. It is worth highlighting that small RNAs, especially piRNAs, are remarkably present in human blood, suggesting their importance as biomarkers for proliferative disorders.^22^

The results also suggested a wide repertoire of Bgl-miRNAs by searching for mature and precursor sequences of miRNAs on the *B. glabrata* genome [Supplementary data **(Table II)**] as previously evidenced.^6^ We identified 89 mature Bgl-miRNAs related to 68 Bgl-miRNA precursors (Table II), some of which are conserved in metazoan, such as bgl-bantam, bgl-let-7, bgl-miR-100, bgl-miR-184, bgl-miR-71, bgl-miR-1984, bgl-miR-1a, bgl-miR-315, bgl-miR-375, bgl-miR-750, bgl-miR-8 and bgl-miR-92.

The bgl-miR-100 had the highest number of reads aligned (4 million reads) onto the without shell snail library, while bgl-miR-1a had more than 2 million reads aligned against both snail libraries (with and without shell). The miR-100 seems to be involved in controlling cell growth,^23^ as well as in the immune system activity.^24^ The large abundance of bgl-miR-100 is even more significant since its analogous miR-100 takes part in the regulation of metabolism and longevity of the *Drosophila melanogaster*.^25^ In *B. glabrata*, the bgl-miR-100 has E3 ubiquitin ligase as a target, and other genes involved in cell biology regulation^26^ and lipids and sugar metabolism [Supplementary data **(Table III)**].

The bgl-miR-1a also seems to be related to cell growth regulation; acting as a tumor suppressor^27^ and regulating heart development in mammalians.^28^ It may affect the development of somatic muscle cells by interfering with E3 ubiquitin ligase activity.^29^ The targets prediction on *B. glabrata* 3’ UTR showed that bgl-miR-1a can regulate ubiquitin proteins, and targets related to muscular organisation, such as actin and fibril in, corroborating with findings in literature [Supplementary data **(Table III)**]. It is also interesting to have observed a significant number of predicted targets on *B. glabrata* are involved in response to stress, which may be determinants of the snail adaptive process to the constant colonisation by numerous microorganisms. In summary, the increased expression ofbgl-miR-100 and bgl-miR-1a evidenced in our study, may play an important role in regulating the development of *B. glabrata* and response to infection.

The remaining miRNAs identified did not show such meaningful abundance, however, they stand out by their probable importance to *B. glabrata* based on their recognised functions in the biology of other organisms as seen in bantam. This miRNA has been reported to play a role in controlling the brain cell growth and feedback regulation of the Notch signaling pathway.^30^ It is suggested the bgl-bantam acts on the development of neuronal cells, mainly in those of tentacle ganglion, a region with dense olfactory neuropils distribution, where there is regular contact of snails with the environment.^31^ In *B. glabrata*, bantam showed a huge difference between the abundance of aligned reads in snail samples without shell (113,854) and with shell (1,307). The targets for this miRNA in *B. glabrata* suggest it can act in regulating the function of the neuronal cell by silencing synapse associated genes. We further suggest a function for bgl-bantam in the regulation of snail metabolism, since it recognises targets with redox function, also with regulatory action over the ubiquitin-protein complex [Supplementary data **(Table III)**].

The mature bgl-miR-71 presented 10,395 reads aligned onto the libraries of snail without shell against337,118 reads onto libraries of those with shell thus, suggesting its higher abundant in the hemolymph. Previous studies have suggested that, in *Caenorhabditis elegans*, miR-71 is involved in longevity by functioning mainly in the nervous system by helping in the maintenance of the proper responses to oxidative stress and thermal shock.^32^ In *Marsupenaeus japonicus*, this miRNA regulates the activation of apoptosis and downregulates phagocytosis, and the pro-phenoloxidase system, indicating its function in the innate immune system of invertebrates.^33^ In *B. glabrata*, the genes RAD50-like for DNA repair, E3 ubiquitin-protein ligase UBR3-like and toll-like receptor 3 [Supplementary data **(Table III)**] were predicted as targets tobgl-mir-71, suggesting it may be important in regulation of mollusk immune system.

The bgl-miR-184 has been linked to a different sort of cellular mechanisms in vertebrates and invertebrates organisms. In *M. japonicus*, this mature miRNA may regulate phagocytosis positively,^33^ similar to our results of target prediction do it. Moreover, in crustacean infected with the virus of white spot syndrome, miR-184 was overexpressed when compared to non-infected one^34^ suggesting its participation in organism immune defense. The bgl-miR-184 has high counts of reads aligned onto its mature sequence in both samples, which may reflex its importance for the mollusk. According to bgl-miR-184 target prediction, it is possible to predict it may act as a regulator of metabolic enzymes, such as aminoacylase-1A-like and ADP-ribosylation factor-like, and also act as a regulator of apoptosis and proteolysis processes [Supplementary data **(Table III)**], thus modulating aging, like in *D. melanogaster*.^25^

The let-7 miRNA is considered a potential regulator of development in *C. elegans*, starting its expression in the larval stage and extending to adulthood.^35^ In *Anopheles stephensi*, this microRNA is differentially expressed between males and females individuals. Furthermore, in *D. melanogaster*, it is significantly more expressed in pupae than in larvae,^36^ also suggesting its involvement in aging modulation and development of nervous tissue.^25^ The counts of reads aligned to mature sequences of bgl-let-7 in the libraries of snail with shell were hugely higher (over 2 million reads) than those aligned onto the mature sequence of the libraries of snails without shell (156,393 reads). This large difference reflects a higher availability of this miRNA in the hemolymph compared to snail tissues. According to the target prediction, bgl-let-7 probably regulates lipid metabolism by controlling the expression of enzymes such as diacylglycerol lipase. It also shows that miRNA also regulates the proteolysis process mediated by ubiquitin by controlling the activity of enzymes such as ubiquitin carboxyl terminal hydrolase [Supplementary data **(Table III)**].

All the Bgl-miRNAs identified in the present study had more than one predicted gene target on the *B. glabrata* genome, similar to the well-recognised potential of miRNAs to act on several targets.^12^ The two softwares used for miRNA target prediction showed different possibilities, however, only the genes predicted by both were accepted as target for Bgl-miRNAs. Through the inspection of the annotations of predicted target genes, it is reasonable to infer that many biological processes are regulated by Bgl-miRNAs, including reproduction, growth, immune process, cell proliferation, metabolic process, and developmental process (Fig. 3B). These results confirmed that Bgl-miRNAs are probably involved in regulations of biological mechanisms such as development of the snail, as expected of this kind of biomolecule. Considering the fact that some genes which are strictly involved in snail immune response to infections were found as targets for Bgl-miRNAs sequenced, it is normal to predict that these molecules may act in regulating *B. glabrata* susceptibility to infection by *S. mansoni*. The involvement of the molecule in modulation of *B. glabrata/S. mansoni* relationship during infection will be investigated in upcoming studies.

Seven of the identified Bgl-miRNAs markedly conserved at the animal clades had their expression profiles further analysed through RT-qPCR. Their level of expression was assessed through different developmental life stages of *B. glabrata*: 10 days (young and sexual immature snails); 20 days (snails in transition to sexual maturity), and 40 days (snails in the adult stage, with complete sexual maturity).^7^ The gene expression in these three stages was compared to that in the pool of *B. glabrata* egg masses control. The precise degree of sexual maturity of the *B. glabrata* snail is poorly studied and not always consensual and, when explored, the authors prefer measuring the diameter of the shell to differentiate adults from young snails. In the present study, we chose a more precise and coherent approach, considering the counting of days of life as a better parameter to mark stages of snail sexual maturity.

Among the selected Bgl-miRNAs in this study, bgl-miR-8 (Fig. 4A) was up-regulated at 10 days compared to its level of expression at other times. At this age, the snail presents intense body growth, suggesting that bgl-miR-8, somehow, regulates biological processes that regulate the development of the snail. Similarly, in *Drosophila* sp. miR-8 acts on the cell surveillance and epithelial organisation of various regulators of act in protein,^37^ thus interfering with the process of homeostasis in the organism.^38^ These findings corroborate our targets prediction for this miRNA, which shows the regulation of processes like metabolism, response to stress and cell organisation [Supplementary data **(Table III)**], through silencing activity over gene targets including E3 ubiquitin-ligase, universal stress protein YxiE-like, TNF receptors, MAP kinases, and DNA-repair.

Bgl-miR-92 was significantly upregulated at 10 days and down-regulated at 20 days of snail development (Fig. 4B). It was predicted to regulate the response to stress and inflammatory process, by targeting such genes as leukocyte tyrosine kinase receptor-like and toll-like receptor [Supplementary data **(Table III)**]. Its ortholog in *Crassostrea gigas* was similarly shown to be involved in the control of inflammatory process by regulating the expression of tumor necrosis factor (TNF).^39^ This earlier finding and our results allow for prediction of bgl-miR-92 function in regulating prominent immunological process in *B. glabrata*. Further, it is worth emphasising that in our study, the snail development was evaluated at an interval when snail presents intense cell proliferation, which was already recognised being regulated by miR-92 in humans.^40^

The miR-184 showed differential expression relative to the stages of development with marked down-regulation of expression in snails of age 20 days (Fig. 4C). The targets found for bgl-miR-184 [Supplementary data **(Table III)**] corroborated with previous findings that orthologs act in the growth regulation, proliferation and, apoptosis of epithelial cells.^41^ ^,^ ^42^ ^,^ ^43^ ^,^ ^44^

Unlike the down regulation shown in reasonable number of miRNAs in the study, bgl-mi-315, bgl-miR-375 and bgl-miR-750 showed up-regulation through all developmental stages of *B. glabrata* (Fig. 4D, F, G). This profile suggested that the targets for these miRNAs play significant role in *B. glabrata* development. However, further studies are required to establish their roles on the biology of the mollusc. The target prediction suggested important action on targets like bcl-2 and caspase-3 [Supplementary data **(Table III)**], well recognised as cell growth regulators. Orthologs of Bgl-miR-315 and bgl-miR-375 act by regulating the cell growth and differentiation in other organisms.^45^ ^,^ ^46^ Since miR-750 and miR-315 seem to play an important role in regulating the innate defense system of shrimp,^47^ our results on targets prediction suggest it has a similar function in *B. glabrata*. We also predicted it as a likely regulator of the piRNAs efficiency in the snail, since it has Piwi and Tudor genes as targets, and ubiquitin-conjugating enzyme E2 D3 [Supplementary data **(Table III)**].

Bgl-miR-1984 (Fig. 4E) was intensely expressed at snail age 10 days. The expression was significantly higher than in other stages. The target prediction showed the regulation of genes related to microtubule activity and metabolic processes linked to immune response [Supplementary data **(Table III)**]. Orthologs of bgl-miR-8 and bgl-miR-1984 were closely related to the regulation of the immune response in *C. gigas*.^48^ This is suggestive of the involvement of miR-1984 in the biology of *B. glabrata* as it is one of the micro RNAs exclusively produced in mollusk. Expression assays using mollusks infected with *S. mansoni* may provide further information about the biological importance of this miRNA.

Through the results described above, the presence of miRNAs in *B. glabrata* was confirmed and their conceivable roles in the biology of the snail were predicted. It is worth mentioning that the existence of the small RNA biogenesis pathway was previously shown, as well as its expression outline in infected and non-infected snails.^7^ The recognition of a well-characterised system for post-transcriptional gene expression regulation system through the Bgl-miRNA opens opportunity for new studies that will aim to elucidate its role in the snail susceptibility to *S. mansoni* infection.

In the present study, we were also able to identify a broad repertoire of Bgl-piRNAs. The role of piRNAs is strongly linked to transposon silencing, epigenetic regulation, and control of mRNA stability in different organisms,^13^ including mollusks.^49^ Our data showed that the putative Bgl-piRNAs identified have strongly conserved uridine at 5´ends (1U) and adenine at the tenth position (10A). It was also identified that most of the sense and antisense reads of Bgl-piRNAs presented an overlap at exactly ten nucleotides in *B. glabrata* genome. This is remarkably a ping-pong cycle signature,^12^ ^,^ ^13^ which shows that *B. glabrata* relies on this biogenesis pathway to generate its mature piRNAs that are able to act as gene expression regulators.

A higher diversity in putative Bgl-piRNAs in snail samples with shell demonstrates that the presence of hemolymph is a determinant to the enrichment of piRNAs repertoire. It is even more relevant since the overall number of aligned reads was higher in the sample without shell. It shows that, even with a smaller depth of sequencing, greater diversity of piRNAs sequences are present in the hemolymph when compared to that of snail tissues.

The relevance of the results in Bgl-piRNAs in *B. glabrata* corroborated with previous studies that showed the presence of piRNAs in circulating fluids of different organisms and are used as biomarkers for several disorders.^22^ The repertoire of circulating Bgl-piRNAs probably regulates different aspects of the snail biology by interfering with a broad range of processes that will be explored in future studies. This is achievable as previous finding shave demonstrated the importance of hemolymph on *B. glabrata* defense against *S. mansoni* infection.^3^ ^,^ ^4^ ^,^ ^5^ ^,^ ^21^

*In conclusion* - This present study showed that *B. glabrata* presented a vast repertoire of miRNAs and piRNAs suggesting that these small RNAs probably interfere with snail biology throughout its life cycle, and act on the immune regulation of this organism. The molecules reported in this study have similar features as those of reference organisms and, present notable diversity, especially in *B. glabrata* hemolymph. The results open possibilities on studying the role of the small RNAs in the mollusk susceptibility to *S. mansoni* infection, as well as its development. This study also provided important information that can be explored for the development of new molluscicides. Additional studies (some already ongoing), involving silencing techniques, may contribute to elucidating functional roles of the small RNAs.
