# Supplementary material for: Deep sequencing of small RNAs reveals the repertoire of miRNAs and piRNAs in Biomphalaria glabrata
Source: Mem Inst Oswaldo Cruz. 2020 Jul 1;115:e190498. doi: 10.1590/0074-02760190498 (PMC7328434; doi:10.1590/0074-02760190498)
Supplement: Supplementary file 1 [file 1678-8060-mioc-115-e190498-s1.pdf]

TABLE I
TaqMan® assays

miRNA	Assay ID	Assay name
miR-8	000269	dme-miR-8
miR-92	241870_mat	dpu-miR-92
miR-184	000290	dme-miR-184
miR-315	000325	dme-miR-315
miR-375	007201_mat	aca-miR-375
miR-750	008337_mat	cte-miR-750
miR-1984	006673_mat	hru-miR-1984
U6 snRNA	001973	U6 snRNA

TABLE II
Genome and transcriptome localisation of *Biomphalaria glabrata* miRNAs

Pre-miRNA	Contig or scaffold	Start	End	Strand	Database
bgl-bantam	LGUN_random_Scaffold555467	2058	2133	minus	Genome
bgl-let-7	LGUN_random_Scaffold446	100296	100363	minus	Genome
bgl-miR-1a	LGUN_random_Scaffold313	141974	142081	minus	Genome
bgl-miR-2a-1	LG6_random_Scaffold1244	15480	15569	plus	Genome
bgl-miR-2a-2	LG6_random_Scaffold1244	16119	16204	plus	Genome
bgl-miR-2b	LG6_random_Scaffold1244	15776	15874	plus	Genome
bgl-miR-2d	LG6_random_Scaffold1244	15674	15762	plus	Genome
bgl-miR-7	LGUN_random_Scaffold1659	33266	33359	plus	Genome
bgl-miR-8	LG3_random_Scaffold555	179139	179237	minus	Genome
bgl-miR-9a	LGUN_random_Scaffold370	127677	127751	minus	Genome
bgl-miR-9b	LGUN_random_Scaffold370	127677	127751	plus	Genome
bgl-miR-12	LGUN_random_Scaffold18	762312	762388	plus	Genome
bgl-miR-29a	LGUN_random_Scaffold532	101201	101301	minus	Genome
bgl-miR-29b	LGUN_random_Scaffold532	117297	117383	minus	Genome
bgl-miR-33	LGUN_random_Scaffold1923	29991	30077	plus	Genome
bgl-miR-34	LG26_random_Scaffold796	58910	58997	plus	Genome
bgl-miR-36a	LGUN_random_Scaffold551705	952	1043	plus	Genome
bgl-miR-67-1	LGUN_random_Scaffold175600	277	358	minus	Genome
bgl-miR-67-2	LGUN_random_Scaffold6774	14221	14302	minus	Genome
bgl-miR-71	LG6_random_Scaffold1244	15354	15442	plus	Genome
bgl-miR-72	LG24_random_Scaffold169	50436	50520	plus	Genome
bgl-miR-87b-1	LGUN_random_Scaffold538	17530	17635	minus	Genome
bgl-miR-87b-2	LGUN_random_Scaffold538	21935	22033	minus	Genome
bgl-miR-92	LGUN_random_Scaffold36250	1201	1300	minus	Genome
bgl-miR-92a	LGUN_random_Scaffold4152	19817	19905	minus	Genome
bgl-miR-92a-1	LGUN_random_Scaffold4152	20211	20307	minus	Genome
bgl-miR-92b	LGUN_random_Scaffold36250	103	192	minus	Genome
bgl-miR-96a	LGUN_random_Scaffold45	468626	468699	plus	Genome

Pre-miRNA	Contig or scaffold	Start	End	Strand	Database
bgl-miR-96b	LG4_random_Scaffold36	489389	489476	plus	Genome
bgl-miR-100	LGUN_random_Scaffold446	93669	93762	minus	Genome
bgl-miR-124	LGUN_random_Scaffold540242	3321	3417	minus	Genome
bgl-miR-125	LGUN_random_Scaffold446	104272	104360	minus	Genome
bgl-miR-133	LGUN_random_Scaffold313	111956	112046	minus	Genome
bgl-miR-137	LGUN_random_Scaffold162	214432	214517	minus	Genome
bgl-miR-153	LGUN_random_Scaffold671	116601	116698	minus	Genome
bgl-miR-184	LGUN_random_Scaffold26723	1552	1675	plus	Genome
bgl-miR-190	LG2_random_Scaffold42	62684	62774	minus	Genome
bgl-miR-193	LG2_random_Scaffold42	420501	420584	minus	Genome
bgl-miR-199a	LGUN_random_Scaffold26964	1961	2054	minus	Genome
bgl-miR-216a	LGUN_random_Scaffold18	759287	759363	plus	Genome
bgl-miR-216b	LGUN_random_Scaffold18	757131	757202	plus	Genome
bgl-miR-219a	LGUN_random_Scaffold276	211507	211604	minus	Genome
bgl-miR-252a	LGUN_random_Scaffold20250	2987	3085	minus	Genome
bgl-miR-252b	LGUN_random_Scaffold7055	558	648	minus	Genome
bgl-miR-277a	LG26_random_Scaffold796	46625	46724	plus	Genome
bgl-miR-278	LGUN_random_Scaffold5083	7879	7980	plus	Genome
bgl-miR-279	LGUN_random_Scaffold3355	16180	16272	plus	Genome
bgl-miR-281	LG24_random_Scaffold115	29213	29283	plus	Genome
bgl-miR-315	LGUN_random_Scaffold550284	2385	2464	minus	Genome
bgl-miR-317	LG26_random_Scaffold796	31870	31967	plus	Genome
bgl-miR-375	LGUN_random_Scaffold682	129296	129395	minus	Genome
bgl-miR-745a	LGUN_random_Scaffold1067	13642	13735	minus	Genome
bgl-miR-745b	LGUN_random_Scaffold1067	13253	13328	minus	Genome
bgl-miR-750	LGUN_random_Scaffold448	178337	178436	minus	Genome
bgl-miR-981	LGUN_random_Scaffold2747	35119	35200	plus	Genome
bgl-miR-1175	LGUN_random_Scaffold448	170932	171027	minus	Genome
bgl-miR-1260	LGUN_random_Scaffold121	296099	296174	plus	Genome
bgl-miR-1984	LG3_random_Scaffold688	139863	139942	minus	Genome
bgl-miR-1985	LG3_random_Scaffold688	136038	136125	minus	Genome
bgl-miR-1986	LGUN_random_Scaffold828	164246	164322	plus	Genome
bgl-miR-1990	LGUN_random_Scaffold828	160164	160258	plus	Genome
bgl-miR-1991	LGUN_random_Scaffold2012	1834	1928	plus	Genome
bgl-miR-1992	LGUN_random_Scaffold126	219037	219133	minus	Genome
bgl-miR-1993	LGUN_random_Scaffold24466	814	884	minus	Genome
bgl-miR-1994a	LGUN_random_Scaffold2100	47010	47095	minus	Genome
bgl-miR-1994b	LGUN_random_Scaffold2100	46368	46454	minus	Genome
bgl-miR-2001	LGUN_random_Scaffold7055	11299	11398	minus	Genome
bgl-miR-2722	LGUN_random_Scaffold2019	53436	53507	plus	Genome

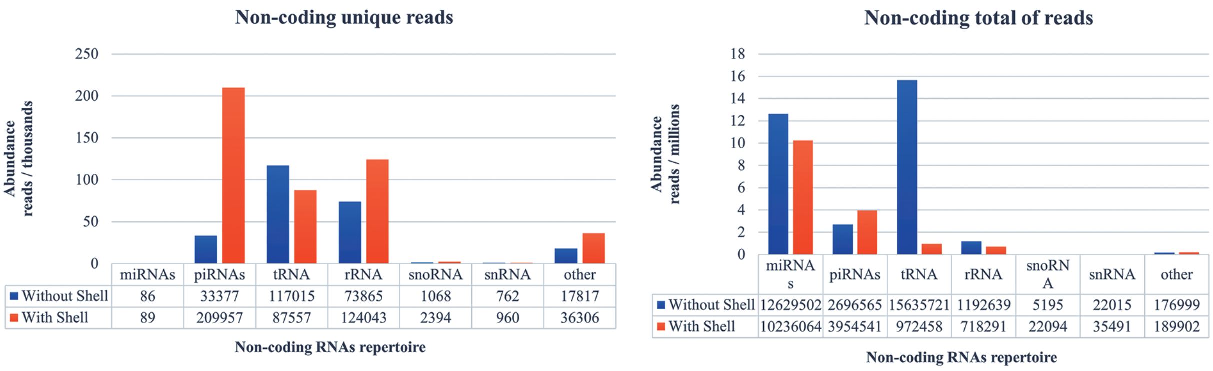

	miRNAs	piRNAs	tRNA	rRNA	snoRNA	snRNA	other
Without Shell	86	33377	117015	73865	1068	762	17817
With Shell	89	209957	87557	124043	2394	960	36306

	miRNAs	piRNAs	tRNA	rRNA	snoRNA	snRNA	other
Without Shell	12629502	2696565	15635721	1192639	5195	22015	176999
With Shell	10236064	3954541	972458	718291	22094	35491	189902

Fig. 1: profile of small RNAs of *Biomphalaria glabrata* compared to Rfam database. (A) unique reads for *B. glabrata* small RNA sequencing with and without shell. (B) total of reads for *B. glabrata* small RNA sequencing with and without shell.

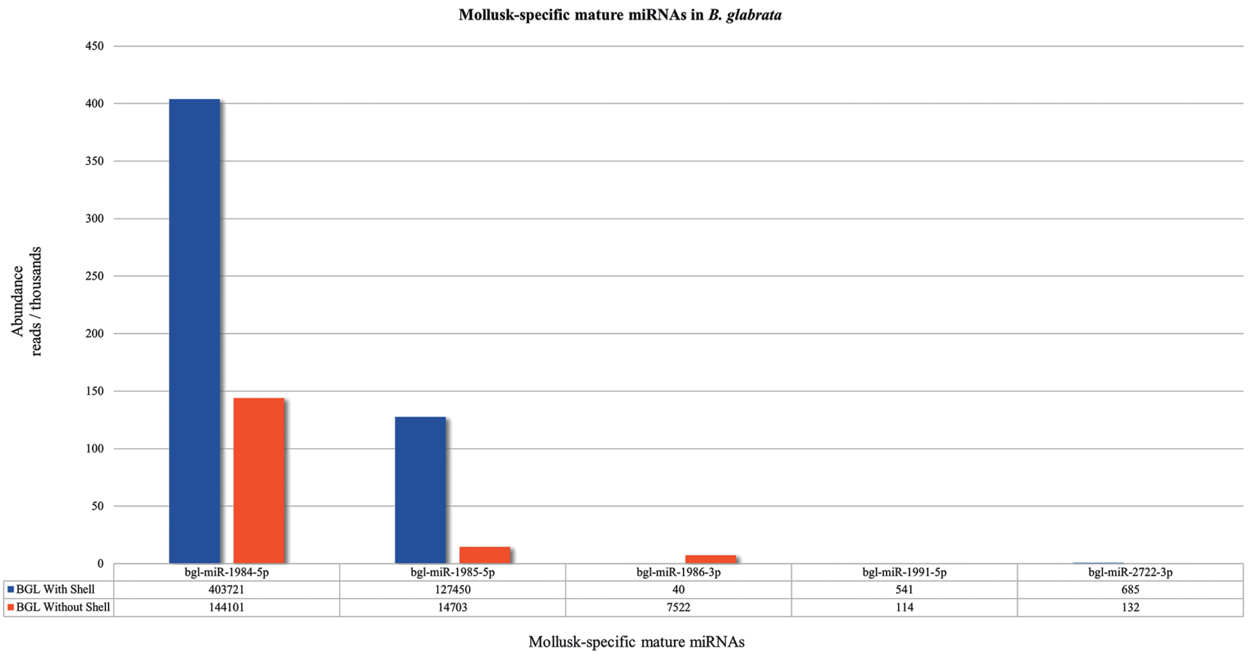

	bgl-miR-1984-5p	bgl-miR-1985-5p	bgl-miR-1986-3p	bgl-miR-1991-5p	bgl-miR-2722-3p
BGL With Shell	403721	127450	40	541	685
BGL Without Shell	144101	14703	7522	114	132

Fig. 2: characterisation of mollusk-specific miRNAs in *Biomphalaria glabrata*. The abundance of mature miRNAs shows a large difference between samples with and without shell, except for bgl-miR-1986-3p that is more representative on mollusks without shell.

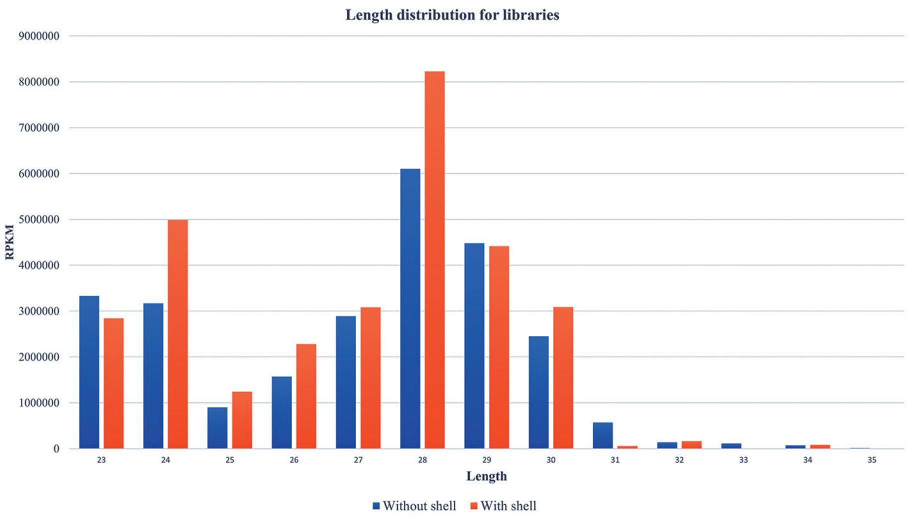

Fig. 3: length distribution at the range of Bgl-piRNAs for small-RNAseq libraries. The libraries of small-RNAseq of *Biomphalaria glabrata* filtered at the range of 23 to 35 nucleotides with large distribution of the reads of 28 and 29 nucleotides.

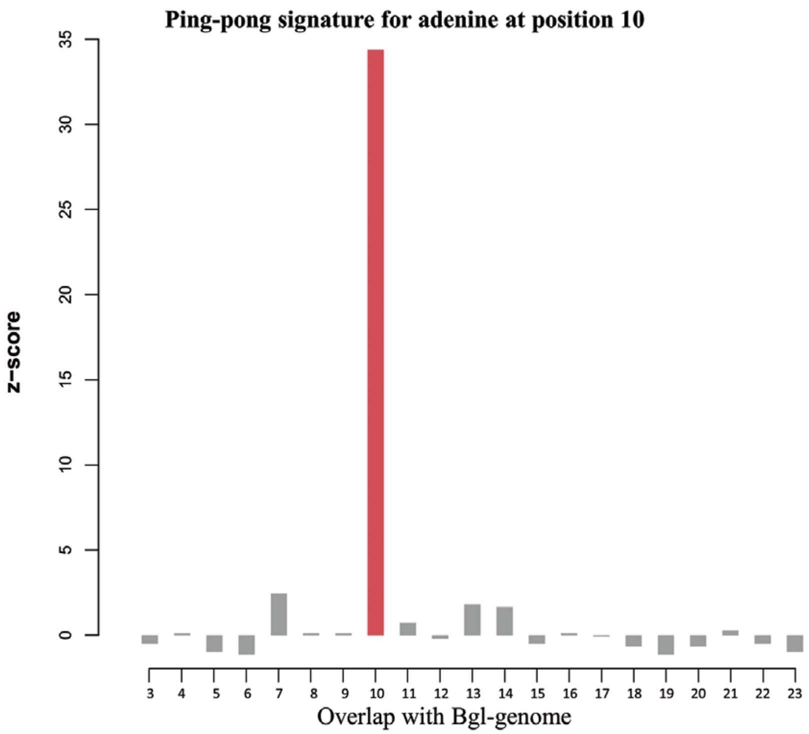

Fig. 4: ping-pong signature for putative Bgl-piRNAs with genome of *Biomphalaria glabrata*. Strong signature of ping-pong cycle with high score for overlapping of Bgl-piRNAs on *B. glabrata* genome with adenine at tenth position.
